# A High-Efficiency Driver Circuit for a Gas-Sensor Microheater Based on a Switch-Mode DC-to-DC Converter

**DOI:** 10.3390/s20185367

**Published:** 2020-09-19

**Authors:** Tzu-Sen Yang, Jin-Chern Chiou

**Affiliations:** Department of Electrical and Computer Engineering, National Chiao-Tung University, 1001 Ta Hseuh Rd., Hsinchu City 30010, Taiwan; samyang80.eed03g@nctu.edu.tw

**Keywords:** microheater, gas sensor, switch-mode converter, low power consumption, Internet of Things

## Abstract

Low power consumption is one of the critical factors for successful Internet of Things (IoT) applications. In such applications, gas sensors have become a main source of power consumption because energy conversion efficiency of the microheater is relative over a wide range of operating temperatures. To improve the energy-conversion efficiency of gas-sensor microheaters, this paper proposes integrated switch-mode DC-to-DC power converter technology which we compare with traditional driving methods such as pulse-width modulation and the linear mode. The results indicate that energy conversion efficiency with this proposed method remains over 90% from 150 °C to 400 °C when using a 3.0, 4.2 and 5.0 V power supply. Energy-conversion efficiency increases by 1–74% compared with results obtained using the traditional driving methods, and the sensing film still detects alcohol and toluene at 200 °C and 280 °C, respectively, with high energy conversion efficiency. These results show that the proposed method is useful and should be further developed to drive gas-sensor microheaters, and then integrated into the circuits of the complementary metal-oxide-semiconductor micro electro mechanical systems (CMOS-MEMS).

## 1. Introduction

**Figure 1:**
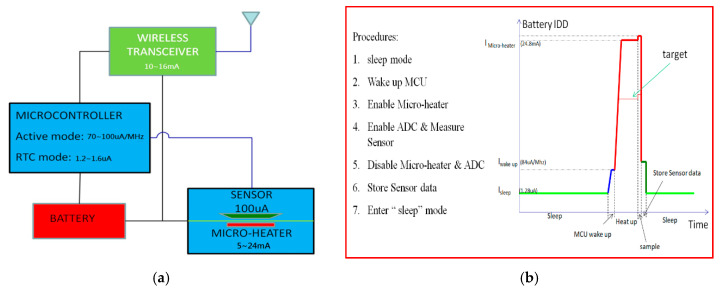
(**a**) Basic block diagram of IoT sensor network and (**b**) operating procedure for IoT sensor network.

Wireless sensors provide crucial information in applications such as the monitoring of environmental conditions or of industrial plants and machinery. As such sensors are easy to install they can be deployed in a variety of situations [1,2,3,4]. However, one of the factors that most restricts the use of wireless sensors is their limited power supply. Figure 1a shows a basic block diagram of a sensor network for an element of the Internet of Things (IoT). Within an IoT sensor network, the sensor’s microheater draws the maximum current. Microcontrollers in low-power technologies are now very mature [5,6] and provide an active operating mode (84 µA/MHz) and several software-selectable low-power operating modes to save battery power. When a microcontroller enters sleep mode, all functions are disabled and only the real-time clock control circuit continues to function (1.28 µA). Wireless transceiver devices with ultralow power consumption are designed and commercialised for IoT applications [3]. 

Figure 1b shows the operating procedure of the sensor network of Figure 1a. The real-time clock control circuit wakes the microcontroller from sleep mode, following which the microcontroller performs its tasks. The microcontroller activates the microheater which controls the sensor temperature. The microcontroller’s analogue-to-digital converter (DAC) samples the sensor output value, then the microcontroller calculates the result as rapidly as possible and stores it in its memory. The goal of the present work is to improve power efficiency to reduce the battery current of active microheaters.

Many microheater studies have focused on designing a smaller heater gas-sensor area to reduce power consumption. However, this increases the importance of maintaining a uniform temperature while sensing and reduces not only the response time for detecting the target gas but also the reliability. Although results may be promising [7,8,9,10,11,12,13], gas-sensor microheaters remain unsatisfactory on the system level. Other gas sensor technologies can be selected, such as nanowire [14,15] or optical gas sensors [16], but the MEMS base is still the majority on the market.

A given type of gas sensor can operate at different temperatures for different gases [17,18]. For example, for ZnO-based gas sensors, the best working temperature for ethanol is 375 °C, whereas it is 200 °C for p-chlorobenzene.

In the past, driving gas-sensor microheaters mainly involved directly controlling the current input to the microheater from the power supply [4,19,20,21,22], and microheaters were mainly operated in pulse-width modulation (PWM) or linear control mode. PWM acts as an on/off switch and the current is supplied only during the ‘on’ part of the duty cycle. The microheater mainly controls the temperature, which is adjusted using the linear control method to set the voltage across the microheater. Generally, this is done by using an OpAmp buffer or linear regulator. Although these traditional methods to drive microheaters are easy to realise and operate over a wide temperature range, their electrical-to-thermal energy conversion efficiency is poor.

In recent years, the development of switch-mode power conversion has matured significantly [23]. This can be realized either inductively [24,25,26] or by using a switched capacitor [27,28,29] and allows the microheater voltage to be controlled. When relatively little heat is generated by the circuit, the conversion efficiency is high. The switch-mode DC-to-DC converter is generally used for power supplies, and the Vout output voltage is fixed. It is not feasible to drive the microheater since the temperature of the microheater cannot be adjusted and there is no related reference design for switch-mode DC-to-DC converter applied to the microheater.

Based on the mentioned drawbacks, in this work we use switch-mode DC-to-DC power conversion to drive the gas-sensor microheater and maintain high energy conversion efficiency over a wide temperature range.

## 2. Proposed Method

**Figure 2:**
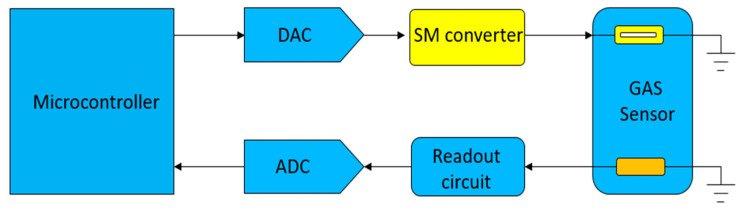
Architecture of proposed system.

As shown in Figure 2, the drive circuit of the gas-sensor microheater uses switch-mode DC-to-DC conversion and we replace the traditional PWM or linear driver to control the power supply current flow with the proposed driver. The traditional driving method is analogous to adjusting a variable resistor between the power supply and the microheater. Although easy to implement, the disadvantage lies in poor energy conversion efficiency. To calculate and measure the energy conversion efficiency η, we use

η = (*V*_out_*I*_out_)/(*V*_in_*I*_in_) × 100% = (*P*_out_/*P*_in_) × 100%,

*V*_out_ = *R*_heater_*I*_out_,

where *R*_heater_ is the microheater resistance, *I*_out_ is the output current (i.e., the current through the microheater), *V*_out_ is the output voltage (i.e., the voltage across the microheater), *V*_in_ is the input voltage (i.e., the voltage supplied by the power supply) and *I*_in_ is the input current (i.e., the current supplied by the power supply).

Because the traditional gas-sensor microheater is driven like a control valve, the current is controlled by the power supply, so the current provided by the power supply is greater than or equal to the current through the microheater: *I*_out_ ≤ *I*_in_. We assume that *I*_out_ = *I*_in_ in the ideal case.

The input voltage *V*_in_ remains unchanged. When the microheater operates over a wide temperature range, the range of output power *P*_out_ is also very large. At high temperatures, *V*_out_ ≈ *V*_in_, and at low temperatures, *V*_out_ ≪ *V*_in_, which reduces the efficiency. Therefore, we propose to use switch-mode DC-to-DC conversion for the microheater so that the current provided by the power supply is less than the current through the microheater: *I*_in_ ≪ *I*_out_.

Using switch-mode DC-to-DC conversion has the following advantages:

1. High-efficiency energy conversion;
2. Except for the microheater, the system produces little heat;
3. The system can be boosted or reduced to allow the microheater to cover the temperature range of a given application.

### 2.1. Implementation of Proposed Method

**Figure 3:**
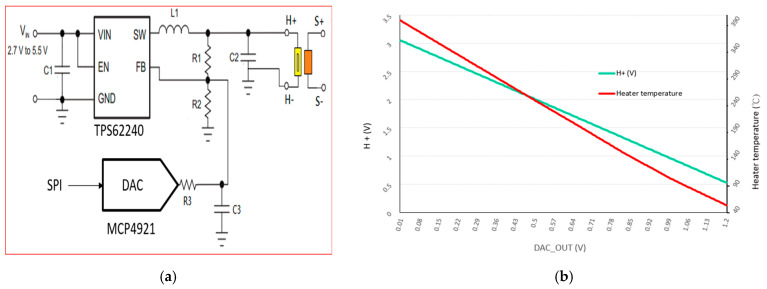
(**a**) Implementation of proposed architecture and (**b**) the microheater temperature and voltage as a function of DAC output voltage.

Figure 3 shows the driving circuit implemented in this paper which consists of a high-efficiency step-down DC-to-DC converter integrated circuit (IC; we used the TPS62240 of Texas Instruments). The input range *V*_in_ of the converter IC is 2.7 to 5.5 V and the output range is adjustable from 0.6 to *V*_in_. The range 2.8 to 4.2 V matches the operating-voltage range of lithium batteries. The output pin SW of the step-down converter IC provides power to the load terminal *H*_+_ through inductor L1 and grounds resistors R1 and R2. Resistors R1 and R2 form a voltage divider and generate a feedback (FB) signal that is sent to the FB pin of the converter IC. When the output voltage is low the FB signal will be less than the internal reference voltage of the converter IC, so the latter will automatically adjust the internal power to increase the output voltage. This process continues until the FB voltage is the same as the internal reference voltage of the converter IC. Therefore, the output voltage is given by the ratio R1/R2.

To control the temperature of the microheater, we used Microchip’s MCP4921 IC, which is a 12-bit DAC and has a serial peripheral interface. The DAC output is connected to the FB pin of the TPS62240 through resistor R3. Using Kirchhoff’s current law, we obtained

(1) $$\frac{H_{+}-FB}{R_{1}}+\frac{DAC_{out}-FB}{R_{3}}-\frac{FB-0}{R_{2}}=0$$

(2) $$H_{+}=\frac{FB*\left(R_{1}*R_{2}+R_{2}*R_{3}+R_{1}*R_{3}\right)-DAC_{out}\left(R_{1}*R_{2}\right)}{R_{2}*R_{3}}$$

Equation (2) shows that the converter output voltage at *H*_+_ (see Figure 3a) decreases linearly with the DAC output voltage, as shown by the green line in Figure 3b.

We used a thermal imager to determine the relationship between the microheater temperature and the input voltage at *H*_+_, and we used Equation (2) to calculate the microheater input voltage *H*_+_ and the microheater temperature as a function of the DAC output voltage (see Figure 3b).

### 2.2. Fabrication of MEMS-Based Microheater

**Figure 4:**
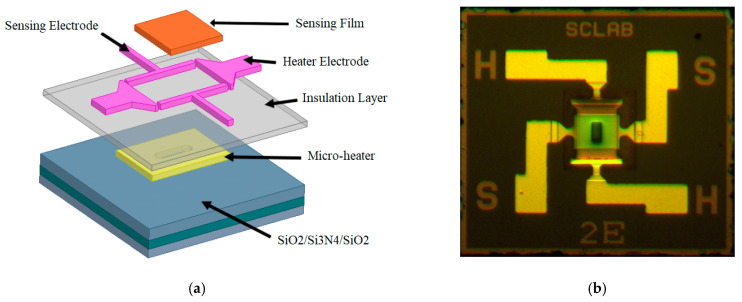
(**a**) MEMS-based microheater structure and (**b**) image of MEMS-based microheater.

The MEMS-based microheater consists of a TaN-based microheater, platinum electrodes, and a stress-free SiO_2_/Si_3_N_4_/SiO_2_ (O/N/O) diaphragm, as shown in Figure 4. The micro-hotplate is 1000 μm × 1000 μm and the TaN microheater is 150 μm × 100 μm. The microheater is enclosed by a membrane with an edge length that is a major factor in determining the heat conduction of the microheater.

### 2.3. Laboratory Environment

Before using the proposed method for gas measurements, we obtained the relationship between the power supplied to the microheater and its temperature, the microheater temperature as a function of DAC_OUT, the power consumed by the microheater as a function of the power provided by the power supply and the stability of the microheater temperature.

**Figure 5:**
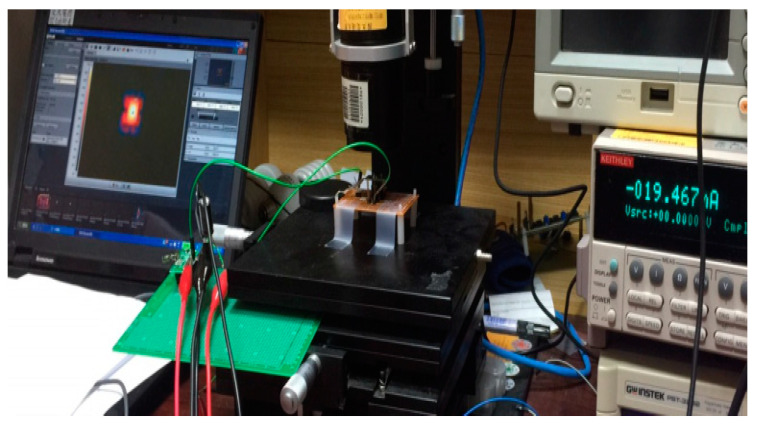
Experimental apparatus to measure parameters of the microheater circuit.

To this end, we used the apparatus shown in Figure 5 to measure the microheater temperature and power consumption. The thermal imager detected the temperature of the microheater to determine the relationship between DAC output and the microheater temperature. The current through the microheater was measured by using an ammeter, and the oscilloscope served to measure the voltage at *H*_+_.

## 3. Results

### 3.1. Onset Speed and Temperature Stability

**Figure 6:**
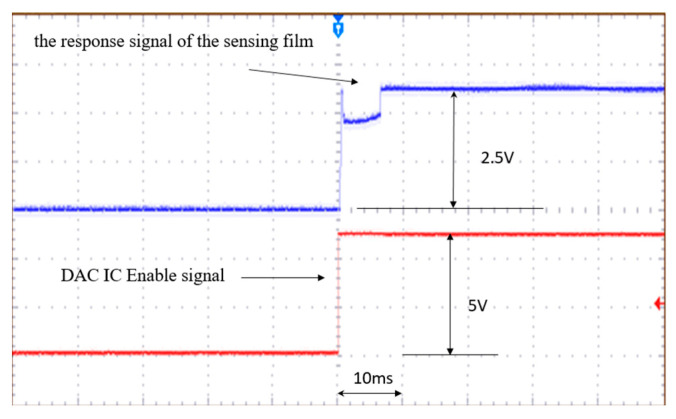
Reaction time of the microheater for proposed driving method. Red line is the DAC Enable signal and blue line is proportional to the response of the sensing film.

In IoT applications, gas detection is typically a short-term operation that strives to conserve power. The pause interval is set by the software. The gas sensor may require a long start-up time before the microheater brings it to its working temperature, and any increase in start-up time will increase power consumption. As shown in Figure 6, the proposed method allows the microheater to quickly reach the operating temperature (the red line is the DAC IC Enable signal and the blue line is proportional to the response of the sensing film). The gas-sensor start-up process requires less than 10 ms.

**Figure 7:**
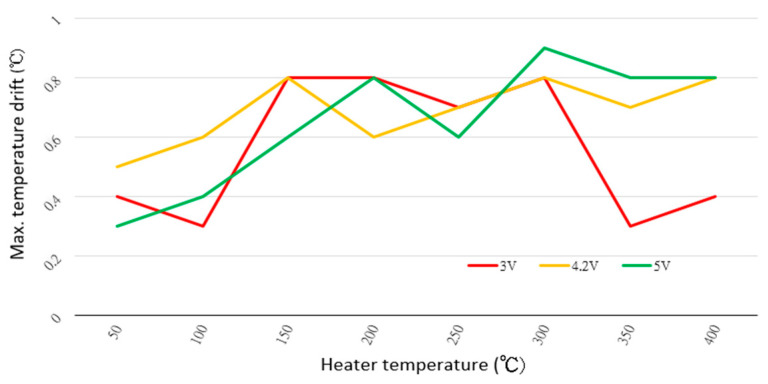
Maximum temperature drift of the microheater (=maximum temperature minus minimum temperature) as a function of the microheater temperature and for several power-supply voltages.

An unstable microheater operating temperature will affect the stability of the gas-sensing film and the measurement results. We set the power supply to 3.0, 4.2 and 5 V to heat the microheater in the proposed circuit. From 50 to 400 °C in 50 °C increments, we recorded the maximum temperature drift (=maximum temperature minus minimum temperature); see Figure 7. Over the entire temperature range measured, the maximum temperature drift is less than 1 °C.

### 3.2. Comparison with Traditional Driving Methods

**Figure 8:**
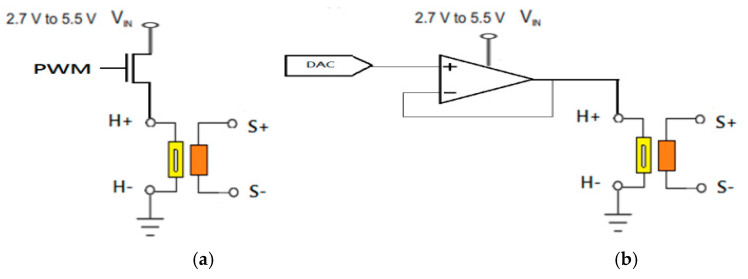
Traditional driver circuit for a gas-sensor microheater: (**a**) PWM driver, (**b**) linear driver (buffer circuit).

We next compared the proposed microheater driving method with traditional PWM and linear driving methods, which are shown in Figure 8. The same microheater is used in the three methods. The central temperature of the microheater is detected by using the thermal imager and recorded as a function power supply voltage (*V*_in_). The current flowing through the microheater alters the operating temperature.

**Figure 9:**
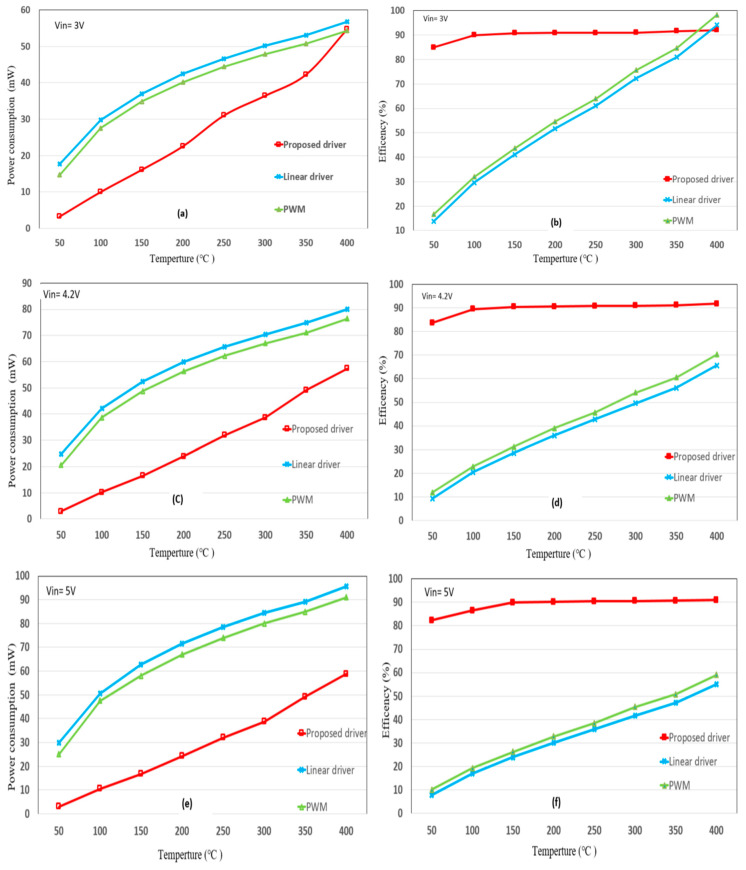
Comparison of power consumption and energy-conversion efficiency between the proposed method and the traditional method: (**a**,**b**) *V*_in_ = 3 V, (**c**,**d**) *V*_in_ = 4.2 V, (**e**,**f**) *V*_in_ = 5 V.

Figure 9a,b compares the power consumption and energy-conversion efficiency of the proposed driver with the same for the traditional PWM and linear drivers for a lithium battery power supply at 3 V and under relatively low power. The driver raises the microheater temperature from 50 °C to 400 °C. As indicated by the figure legend, the curves show the power consumption and energy conversion efficiency of the proposed driver, PWM driver and the linear driver. When the microheater is between 350 °C and 400 °C, the energy conversion efficiency of each of the three driving methods exceeds 80%. However, below 300 °C the energy-conversion efficiency of the proposed method is significantly greater than that of the traditional driving methods; with the proposed method, the microheater maintains an energy-conversion efficiency of 85% to 90% from 100 °C to 400 °C.

For Figure 9c,d, the lithium battery is fully charged at 4.2 V output voltage. The proposed driver method maintains the same power consumption as when the lithium battery operates at 3 V, whereas the power consumption increases for the traditional driving methods. For example, when the microheater is driven to 400 °C, the power consumption of the three methods is about 55 mW when the power supply is at 3 V. For the proposed driving method, this power consumption remains the same when the power supply is at 4.2 V, but the power consumed by traditional methods increases to over 75 mW.

Figure 9e,f consider the use of a universal serial bus 5-V power supply. When the microheater is below 300 °C, the proposed method consumes about half of the power consumed by traditional methods. The conversion efficiency of the traditional driving method is only about 55% at 300 °C, as opposed to over 90% for the proposed driving method.

### 3.3. Detecting Gas with Proposed Method

**Figure 10:**
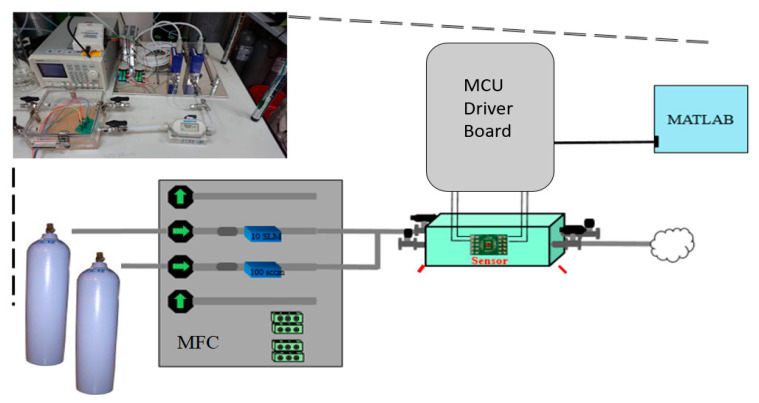
Photograph and schematic illustration of gas-detection test environment.

Figure 10 shows the experimental apparatus for detecting gas. The mass flow controller (MFC) is used to adjust the toluene and alcohol concentration. The MCU driver board is used to control the microheater temperature. We used an oscilloscope to monitor the resistance of the sensing film. Ambient air was allowed to enter the apparatus first, following which the microheater was driven to 300 °C, then 220 °C.

**Figure 11:**
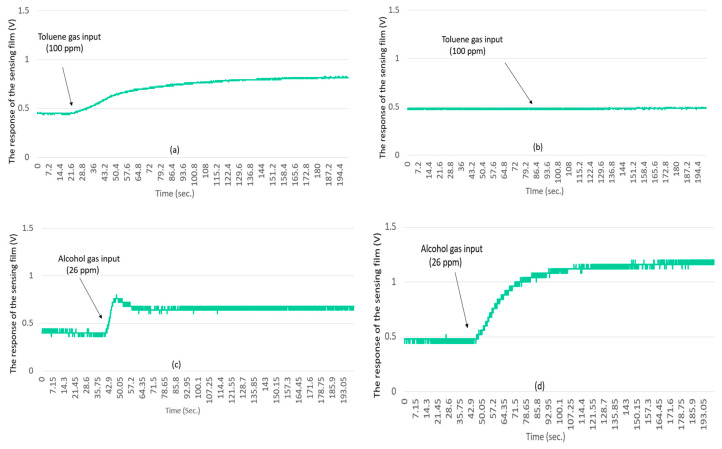
Resistance of gas-sensor film for (**a**) proposed driving method applying 34 mW to the microheater and injecting 100 ppm toluene; (**b**) proposed driving method applying 26 mW to the microheater and injecting 100 ppm toluene; (**c**) proposed driving method applying 34 mW to the microheater and injecting 26 ppm alcohol; (**d**) proposed driving method applying 26 mW to the microheater and injecting 26 ppm alcohol.

For Figure 11a, the proposed driver circuit drove the microheater to 280 °C, then the MFC injected 100 ppm toluene in essentially a step-function fashion. The oscilloscope directly monitored the voltage between the sensing film and the reference resistor. The resistance of the sensing film changed significantly upon injecting 100 ppm toluene. Figure 11b shows the same experiment but with the microheater at 200 °C. This time, the resistance of the sensing film did not change. Figure 11c shows the same experiment as Figure 11a, but with 26 ppm alcohol injected as a step function. Upon injecting the alcohol, the resistance of the sensing film dropped sharply and then rose again, so the alcohol measurement result was not ideal. Finally, Figure 11d shows the results of the same experiment as Figure 11c, but with the microheater at 200 °C. The output signal was similar to that of Figure 11a. These results are consistent with those of Reference [14], which describe the need to drive the microheater to different operating temperatures for different target gases. Thus, the proposed method satisfies the output requirements for operation over a wide temperature range.

## 4. Discussion

The results of Figure 6 and Figure 7 show that the proposed method stably drives the microheater and that the required start-up time is very short. The results of Figure 9 show that, although the proposed method is more complicated than traditional methods, the former maintains higher energy conversion efficiency over a wide range of power input and output.

When using a lithium battery power supply charged at 3 or 4.2 V, the proposed method maintains the same power consumption, especially at medium and low temperatures, which leads to a significantly greater energy conversion efficiency than with traditional driving methods. This increases the battery’s lifetime and reduces the need to recharge, so smaller batteries can be used. In addition, the miniaturisation of the sensor network is very helpful for IoT applications.

Figure 11 shows that the gas sensor must be operated at different temperatures for different target gases. The proposed driving method provides high energy-conversion efficiency over a wide range of operating temperatures and can be used to detect gases at different temperatures.

## 5. Conclusions

**Figure 12:**
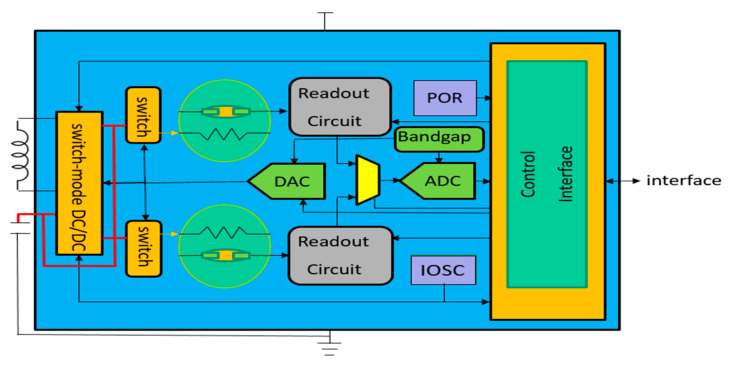
CMOS-MEMS base gas sensors function block.

The integrated switch-mode DC-to-DC converter technology is very useful for driving gas-sensor microheaters. For IoT applications in particular, this technology functions over the wide range of power input and working temperatures of the various target gases. It also maintains high energy-conversion efficiency over a wide range of temperatures. Finally, although the microheater-control circuit is slightly more complicated than traditional circuits, it is compensated by its excellent energy conversion efficiency. In the future, such circuits could be integrated into CMOS-MEMS circuits to obtain miniature, high-efficiency gas-sensing devices, as shown in Figure 12.
